# Structural, functional and molecular pathogenesis of pelvic organ prolapse in patient and *Loxl1* deficient mice

**DOI:** 10.18632/aging.203777

**Published:** 2021-12-19

**Authors:** Yu Li, Nanfang Nie, Lin Gong, Fangyuan Bao, Chengrui An, Hongxia Cai, Xudong Yao, Yanshan Liu, Chunbo Yang, Bingbing Wu, XiaoHui Zou

**Affiliations:** 1 Clinical Research Center, The First Affiliated Hospital, School of Medicine, Zhejiang University, Hangzhou, Zhejiang 310003, PR China; 2 Dr. Li Dak Sum and Yip Yio Chin Center for Stem Cells and Regenerative Medicine, School of Medicine, Zhejiang University, Hangzhou, Zhejiang 310003, PR China; 3 Key Laboratory of Tissue Engineering and Regenerative Medicine of Zhejiang Province, Zhejiang University, Hangzhou, Zhejiang 310003, PR China; 4 Department of Gynaecology, The Fourth Affiliated Hospital, School of Medicine, Zhejiang University, Hangzhou, Zhejiang 310003, PR China; 5 Department of Gynaecology, Women’s Hospital, School of Medicine, Zhejiang University, Hangzhou, Zhejiang 310003, PR China

**Keywords:** pelvic organ prolapse, pathogenesis, animal model, Loxl1 knockout mice

## Abstract

Pelvic organ prolapse is a worldwide health problem to elderly women. Understanding its pathogenesis and an ideal animal model are crucial to developing promising treatments. The present study aimed to investigate new clinical significance and detailed mechanism of pelvic organ prolapse by comparing the structural, functional and molecular dysfunctions of pelvic organ prolapse in patient and *Loxl1* deficient mice. Our results showed that human vagina tissues from prolapsed site showed disarranged collagen and elastic fibers compared with the non-prolapse tissue. A gene ontology (GO) analysis of differentially expressed genes revealed molecular changes mainly related to inflammatory response and extracellular matrix (ECM) organization. While the mice lacking *Loxl1* developed stable POP phenotype and disordered ECM structure in histology. Such *Loxl1* knockout mice exhibited a significantly urinary dysfunction and decreased mechanical properties of the pelvic floor tissues, implying that POP in human condition might be induced by progressively decreased mechanics of pelvic tissues following ECM catabolism. Similarly, we not only identified significant up-regulated ECM catabolism processes and down-regulated ECM synthesis processes, but also characterized high level of inflammatory response in vagina tissue of the *Loxl1* deficient mice. Thus, all these pathological changes in the POP mice model was consistent with those of the clinical elderly patients. These findings provide new insight into remodeling of POP by LOXL1 regulation and be of great importance to develop combination treatments of ECM metabolism and inflammation regulation strategy.

## INTRODUCTION

Pelvic organ prolapse [1] refers to the pelvic floor support functional disorder with the female pelvic cavity viscera (such as bladder, uterus, vaginal stump, etc.) moving down along their normal position. The POP symptoms not only seriously disrupt social and daily activities but also influence the mental health of middle-aged and old women [2]. Tradition treatments of POP include conservative treatment and traditional surgery, but the treatment effect is not satisfactory. The recent techniques, including novel biomaterials and cell-based therapies, have been shown to improve quantification and modelling of the biomechanics of the pelvic floor [3]. When new products need a long-term safety evaluation before proceeding to clinical trials, the choice of animal models in this assessment become particularly important.

Several animal species, including non-human primates (NHPs, such as rhesus monkeys, squirrel monkeys, baboons), pigs, cattle and sheep have been studied to spontaneously mimic forms of POP in women [4]. However, these big animals are not conducive for use in laboratory research due to long production period and high cost [5–8]. On the contrary, the rodents, like rats and mice, can easily appear POP phenotype within a short time after gene manipulation. Until now, there are varieties of gene knockout mice exhibiting POP phenotype [9–12]. Among them, Lysyl oxidase like-1 (Loxl1) knockout mice were most widely used as POP mice model. Loxl1 is a key enzyme in maintaining the dynamic homeostasis of elastin fibers [13]. Clinical studies showed decreased expression of Loxl1 and elastin content in women with POP [14, 15]. The Loxl1 knockout mice displays typical phenotype of POP with rectal prolapse [13, 16]. Similar to the prevalence of POP in women, delivery and aging are the leading risk factors in Loxl1 knockout mice, as the prolapse grades and incidences would increase with delivery times and ages [11, 16–18]. According to the pelvic anatomy assessment, Loxl1 knockout mice had greater variability in the size and location of the bladder [11]. In biomechanical behavior, Loxl1 knockout mice showed a 31% reduction in ultimate load at failure [18]. Loxl1 knockout mice also showed lower urinary dysfunction with higher urine frequency of urinary events and decreased leak point pressure (LPP) and urine output per event [11, 16, 19, 20]. To sum up, the validation of Loxl1 knockout mice acting as POP model mainly include that the anatomical and functional phenotype resemble POP in human. However, an ideal animal model not only can simulate POP phenotype of human, but also can simulate the pathogenesis of POP. Whether Loxl1 knockout mice can match the pathology of POP in human remains unclear, and the correlation between human and mice model still warrants further investigation.

To answer this question, we compared the structural and functional dysfunctions of pelvic organ prolapse in patient and *Loxl1* deficient mice, and use RNA-seq to resolve the molecular underpinnings of POP at high resolution, analyzing the transcriptomes of both human and mice samples. Our data: (1) deconvolve the structural, functional and molecular pathogenesis in healthy and prolapsed tissues; (2) reveal the pathological similarity between human POP and Loxl1 knockout mice model. Our work highlights the availability of Loxl1 knockout mice in identifying the molecular pathways of POP driving abnormal ECM homeostasis and inflammatory response and therefore should serve as an ideal model for the high-precision identification of cellular, molecular complexity and related therapeutic targets in POP.

## MATERIALS AND METHODS

### Study design and experimental procedure

Human samples of vaginal tissues from clinical patients with POP were collected from seven women with POP undergoing hysterectomy for benign conditions at Hospital of Obstetrics and Gynecology Affiliated to Medical College of Zhejiang University. A full thickness vaginal wall sample was taken from the POP site and an additional vaginal wall sample was taken from the non-prolapsed site of the vaginal cuff. Inclusion in the POP group required uterine or vagina prolapse beyond the hymen (stage III or IV). The patient demographics and the POP-Q stage were showed in Table 1.

**Table 1:** The patient demographics and the POP-Q stage.

	**Donor 1**	**Donor 2**	**Donor 3**	**Donor 4**	**Donor 5**	**Donor 6**	**Donor 7**
Age	77	64	82	70	72	53	55
BMI	26.56	21.72	20.40	28.40	24.97	23.44	24.24
Gyn history	G5P3	G1P1	G3P3	G3P2	G3P3	G2P1	G2P1
POP-Q stage	III	III	III	IV	IV	III	III

Female C57BL/6 wild type mice and the Loxl1 knockout mice (at the age of 12 month) were kept in a specific pathogen-free air-conditioned room and were allowed free access to food and water at the Animal Center of Zhejiang University of Medicine. All experiments were approved by the Ethics Review Board for Animal Studies of Zhejiang University. Wild type mice (*n* = 6) and the Loxl1 knockout mice (*n* = 7) were used for leak-point pressure (LPP) testing. Wild type mice and Loxl1 knockout mice (*n* = 4 in each groups) were used for mechanical properties of pelvic floor samples. WT mice and Loxl1 knockout mice (*n* = 4 in each group) were used for RNA-seq.

### Ethical statement

All procedures of human samples and animal in this article were performed with ethics approved protocols, in accordance with guidelines of Ethics Committee of College of Medicine, Zhejiang University (Reference number: 2015–181) and The Lab of Animal Experiment Ethical Inspection of College of Medicine, Zhejiang University (Reference number: 2015–112), respectively.

### Histological examination

Specimens were immediately fixed in 4% neutral buffered paraformaldehyde, dehydrated through an alcohol gradient, cleared, and embedded in paraffin blocks. Histological sections (10 μm) were prepared using a microtome, and subsequently de-paraffinized with xylene, hydrated using decreasing concentrations of ethanol and then subjected to hematoxylin and eosin (H&E) staining, Weigarts’ staining (specific staining for elastic fibers) and Sirius Red staining (specific staining for type I and type III collagen fibers). Then the sections were mounted and observed under microscopy.

### Western blotting

Vagina tissues from human and animal were lysed in lysis buffer (150 mM sodium chloride, 50 mM Tris, pH 7.3, 0.25 mM EDTA, 1% (w/v) sodium deoxycholate, 1% (v/v) Triton X-100, 0.2% sodium fluoride, 0.1% sodium orthovanadate, and a mixture of protease inhibitors from Roche Applied Science). Samples were run in SDS/PAGE gels and analyzed by Western blotting with primary antibodies Col1a1 (Affinity, AF7001, 139KDa), Col3a1 (Beyotime, AF6531, 170KDa) and Elastin (HUABIO, ER1908-02, 70KDa).

### Immunofluorescence

The tissues were fixed in 4% (w/v) paraformaldehyde, and then dehydrated in an ethanol gradient, prior to embedment in paraffin and sectioning at 10 μm thickness. Immunostaining were carried out as follows: The 10 μm paraffin sections were rehydrated, antigen retrieved, rinsed three times with PBS, and treated with blocking solution (1% BSA) for 30 min, prior to incubation with primary antibodies at 4°C overnight. The primary antibodies Col1a1 (Affinity, AF7001), Col3a1 (Beyotime, AF6531), Elastin (HUABIO, ER1908-02), CD45 (Biolegend, 368510), CD68 (Beyotime, AF6432) and F4/80 (Invitrogen, 12-4801-82) was used to detect the collagen fiber and elastin fiber deposition. Secondary antibody goat anti-rabbit Alexa Fluor 546 (Invitrogen, A11035) and DAPI (Beyotime, China) were used to visualize the respective primary antibodies and the cell nuclei. All procedures were carried out according to the manufacturer's instructions.

### Leak-point pressure (LPP) testing

LPP testing were performed as described in Li Bing Shi et al. [21]. Wild type mice (*n* = 6) and the Loxl1 knockout mice (*n* = 7) were tested as following procedure. Each anesthetized animals were placed supine at the level of zero pressure and the bladder was emptied manually. Subsequently the bladder was filled with warm saline (5 ml per hour for mice) at room temperature through a Y-type 22G catheter (BD, USA), while the bladder pressure was recorded. One end of the Y-type suprapubic catheter was connected to a Biological Signal Collecting System (RM6240, Chengdu Biological Instruments, Chengdu, China), and the other end was connected to a microinjection syringe pump. All bladder pressures were referenced to air pressure at the level of the bladder. Pressure transducer signals were collected and digitized for data collection. Bladder pressure increased gradually and the peak pressure was recorded when leakage occurred. The pressure at which visible leakage occurred was defined as the LPP, and this was measured 30 min per animal. Values from the 10 first continuous peaks were considered for the study.

### Mechanical property by pelvic floor support testing

Mechanical properties of pelvic floor samples from wild type mice and Loxl1 knockout mice (*n* = 4 in each groups) were evaluated following the protocol reported by previous studies [22]. The compressive mechanical properties of the pelvic floor were tested with an Instron testing machine (model 5543; Instron) and software (Bluehill V2.0; Instron), using a 5 mm diameter cylindrical indenter fitted with a 10N maximum loading cell. The unconfined equilibrium modulus was determined by applying a step displacement (20% strain), and monitoring the compressive force over time until equilibrium was reached. The displacement of the cylindrical indenter was tested to estimate strain for applied deformations. The crosshead speed used was 10 mm/min. The ratio of equilibrium force to the cross-sectional area was divided by the applied strain to calculate the equilibrium modulus (in MPa).

### Atom force microscope (AFM) image

Nanoscale morphology of the vaginal tissues was measured by AFM, using previously established techniques [23]. Briefly, OCT embedded vaginal tissues of wild type and Loxl1 knockout mice were sectioned (30 μm) and mounted on glass coverslips. These AFM samples were allowed to air dry for at least 24 hours before AFM analysis. Nanoscale morphology was determined by NanoScope IIIa AFM system (Bruker, Santa Barbara, CA, USA) using Tapping mode using a silicon AFM probe (OMCL-AC240TS, force constant 1.7N/m, resonant frequency 65–89 kHz,). AFM was conducted at Analysis and Test Center of Shanghai Jiao Tong University.

### RNA-seq

RNA-seq and bioinformatic data analysis were performed by Shanghai Novelbio Ltd. Total RNA was extracted from each sample from vaginal tissues of WT mice and Loxl1 knockout mice (*n* = 4 in each group) by Trizol reagent (Invitrogen) separately. The RNA quality was checked by Bioanalyzer 2200 (Aligent) and kept at −80°C. The RNA with RIN (RNA integrity number) >8.0 is acceptable for cDNA library construction. The cDNA libraries for single-end sequencing were prepared using Ion Total RNA-Seq Kit v2.0 (Life Technologies) according to the manufacturer’s instructions. Then the prepared library was loaded on to 1 P1v2 Proton Chip (Life Technologies) and sequenced on Proton Sequencers according to Ion PI Sequencing 200 Kit v2.0 (Life Technologies). Before read mapping, clean reads were obtained from the raw reads by removing the adaptor sequences. The clean reads were then aligned (version: Mfa5.0) using the MapSplice program (v2.1.6). In alignment, preliminary experiments were performed to optimize the alignment parameters (-s 22 -p 15–ins 6–del 6–non-canonical) to provide the largest information on the AS events [24].

### Statistical analysis and RNA-seq data analysis

The quantified values between groups were assessed using *t*-test to detect differences between pathological and control groups, with statistical significance set at *p* < 0.05. The RNA-seq data analysis was performed by R version 3.3.2 (Platform: i386-w64-mingw32/i386 (32-bit)). And Read alignment and count quantification is conducted using the Rsubread package and the statistical analyses are performed using the edgeR package. The differential expression analysis uses the quasi-likelihood functionality of edgeR.

### Data availability

The gene chip data were collected from GEO dataset as follows: GSE12852, GSE53868. The data in this paper would be publicly available upon publication.

## RESULTS

### The micro-structure changes of vaginal tissue in clinical POP patients

**Figure 1:**
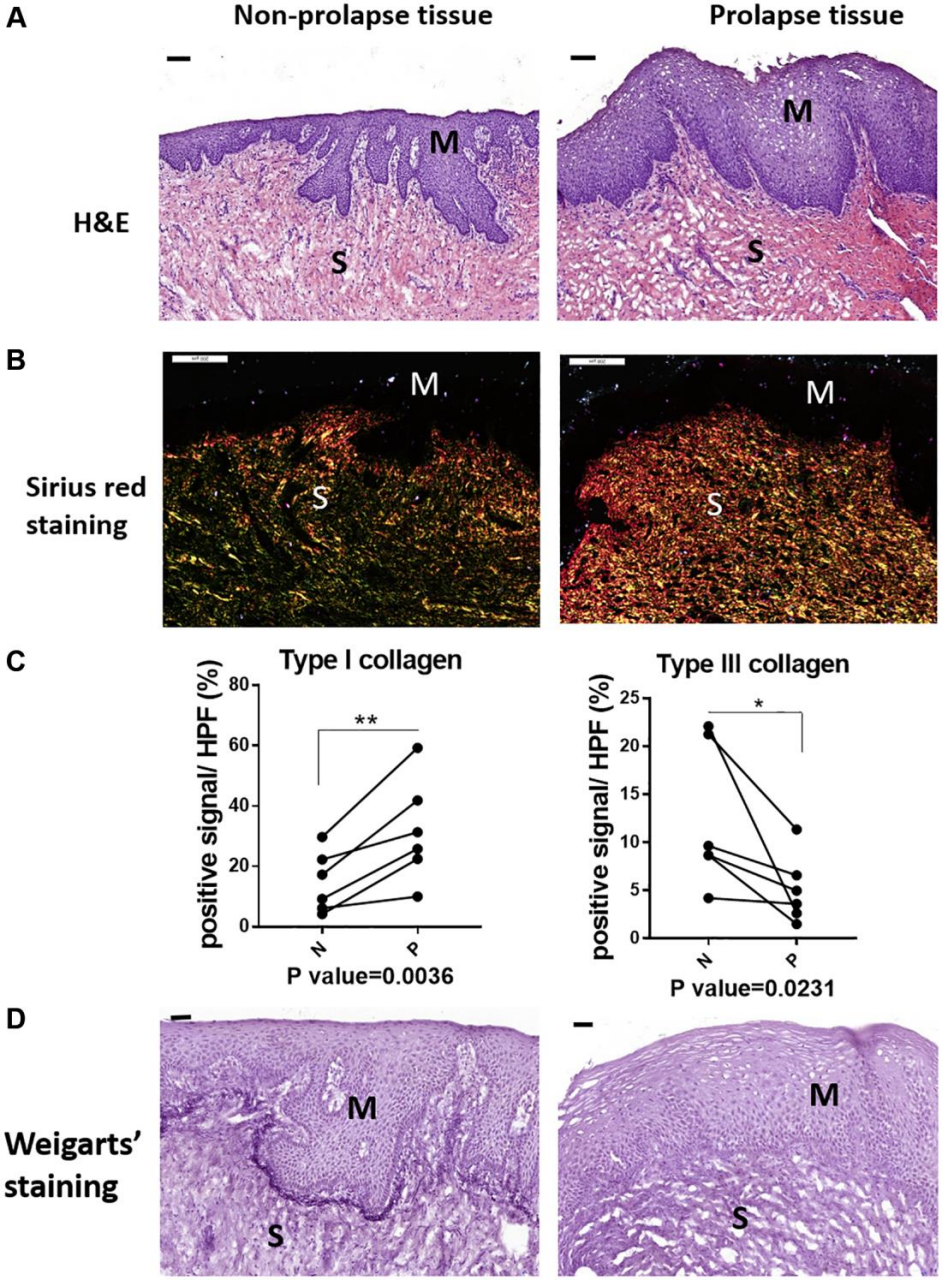
**The micro-structural changes in vagina tissue of pelvic organ prolapsed patients.** (**A**) HE staining revealed obvious microstructure porosity and disappeared Vicious Angle structure in the prolapsed tissue (M: mucous layer, S: submucosa layer. scale bar 100 um), (**B**) Sirius red staining showed increased type I collagen and decreased type III collagen in the prolapsed tissue (type I collagen was red and bright yellow, type III collagen was green, scale bar 200 um), (**C**) Sirius red staining as in B was quantified, the proportion of positive signals area (red signals for type I collage and green signals for type III collagen) in each High Power Field of vision (HPF) were quantified, *n* = 6 in each group. (**D**) Weigarts’ staining demonstrated disappeared elastic fibers near basal layer in the prolapsed tissue (scale bar 50 um).

Prior to identify the pathology of POP, the micro-structural changes of prolapsed tissue from patients were initially investigated. Compared with the normal region by histological staining, prolapse site showed a loose structure in submucosa (Figure 1A). Abundant type I collagen (the red one under polarized light) were observed in the prolapsed region according to Sirius red staining, while the content of type III collagen in green color were decreased apparently (Figure 1B). The quantification result confirmed the significantly increased type I collagen and significantly decreased type III collagen in prolapsed tissues (Figure 1C). The changes of collagen indicated an increased ratio of type I collagen/type III collagen in the prolapsed samples. The Weigarts’ staining (elastic fiber specific staining) showed fragmentation or short rod of elastic fiber in prolapsed tissues, whereas the linear arranged fibers were disappeared. Furthermore, the elastin fibers near basement membrane are in disorder as well (Figure 1D).

**Figure 2:**
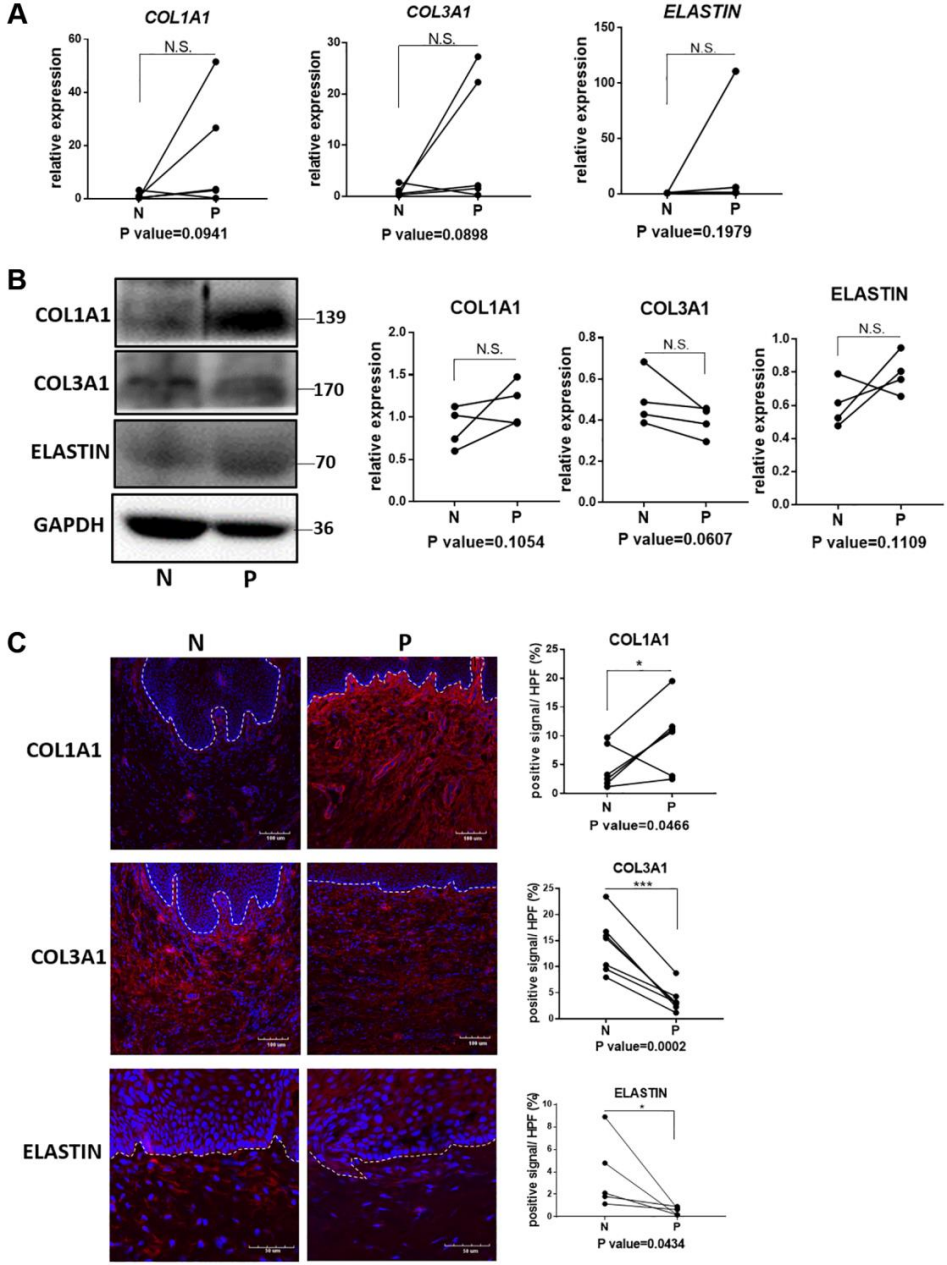
**Quantification analyses of type I collagen, type III collagen and elastin in vagina tissue of pelvic organ prolapsed patients.** (**A**) qRT-PCR analyses showed mRNA expression level of COL1A1, COL3A1 and ELASTIN in vagina tissue (N: non-prolapsed tissue, P: prolapsed tissue, *n* = 4 in each group). (**B**) Western blots analyses of COL1A1, COL3A1 and ELASTIN in vagina tissue. Relative content of each protein were quantified on the right, the levels of protein expressed relative to GAPDH, (*n* = 4 in each group). (**C**) Immunofluorescence staining for visualization (red) of COL1A1, COL3A1 and ELASTIN in vagina tissue (White dotted line indicated the interface between mucus layer and submucosa layer). The proportion of positive signal area (red) in each High Power Field of vision (HPF) of each protein were quantified on the right, (*n* = 6 in each group).

The qRT-PCR analyses showed relatively increased mRNA expression of Col1a1, Col3a1 and elastin in prolapsed tissue of human samples (Figure 2A), which may relate to compensatory gene expression as the disorder of collagen and elastin fiber structures. Western blot and immunofluorescence staining showed increased protein level of col1a1 but decreased level of col3a1 in prolapsed tissues (Figure 2B and 2C). These changes of collagen coincided well with those of quantified in Sirius red staining. Immunofluorescence staining for elastin showed loss of elastin fibers deposition (Figure 2C), with concomitant tropoelastin accumulation (Figure 2B). Overall, the prolapsed vaginal tissue had an abnormal ECM micro-structure and composition.

### The molecular changes in vagina tissues from patients with pelvic organ prolapse

**Figure 3:**
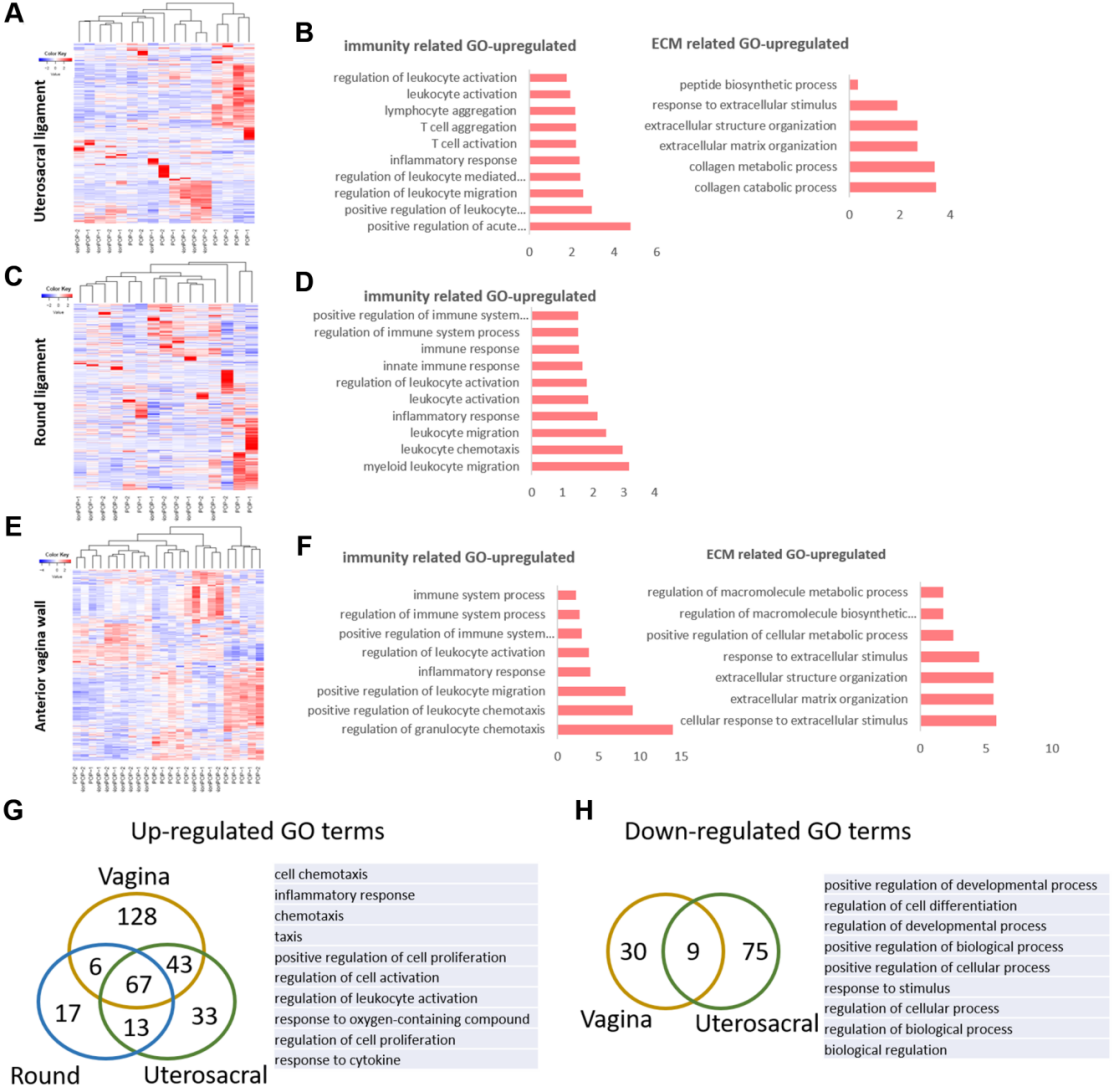
**The molecular changes in pelvic tissue of pelvic organ prolapsed patients.** (**A**, **B**) Heat map across all the samples using the top 500 most differently expressed genes (**A**) and gene ontology (GO) categories (**B**) of up-regulated differently expressed genes in uterosacral ligament between the prolapsed group and non-prolapsed group. (**C**, **D**) Heat map across all the samples using the top 500 most differently expressed genes (**C**) and gene ontology (GO) categories (**D**) of up-regulated differently expressed genes in round ligament between the prolapsed group and non-prolapsed group. (**E**, **F**) Heat map across all the samples using the top 500 most differently expressed genes (**E**) and gene ontology (GO) categories (**F**) of up-regulated differently expressed genes in vagina tissue between the prolapsed group and non-prolapsed group. (**G**) Venn diagram showing the overlaps of up-regulated differentially expressed genes enriched GO terms between vagina, round ligament and Uterosacral ligament, the top 10 GO terms also showed on the right side. (**H**) Venn diagram (left) and the form (right) showed the overlaps of down-regulated differentially expressed genes enriched GO terms between vagina and Uterosacral ligament.

To further understand the possible biological events and potential molecules mechanisms in the pathogenesis of POP, we collected the gene chip data of three different pelvic floor tissues in POP patients from NCBI, including uterosacral ligament, the round ligament of uterus (serial number is GSE12852) [25] and anterior vaginal walls (serial number is GSE53868). The round ligament and the uterosacral ligament of the uterus were derived from 8 POP patients and 9 non-POP patients. The anterior vaginal wall was collected from the prolapsed site and non-prolapsed site in 12 premenopausal women with POP. We got the sample cluster and gene cluster according to the differently expressed genes (Figure 3A, 3C, 3E). From the results of clustering, the samples of round and uterosacral ligament from POP and non-POP patients couldn’t be clustered into two but four categories, indicating a great variation between patients (Figure 3A, 3C). And the anterior vaginal wall from prolapsed site and non-prolapsed site had the similar results of clustering (Figure 3E). Next GO analysis was performed to identify the biological process or molecular signaling pathways in POP development (Figure 3B, 3D, 3F). There were plenty of immune function related GO terms in all three tissues from prolapsed group, such as acute immune response, leukocyte activation and migration, the activation of T cells. Such up-regulated immune related GO terms may represent a high level of inflammation and immune defense in the prolapsed region. In addition, there were up-regulated groups of ECM associated GO terms: ECM organization in uterosacral ligament and vaginal wall, and collagen metabolism and catabolism process in uterosacral ligament (Figure 3B). That indicated an ECM related disordered biological process occurred in the prolapsed samples. To figure out the common pathological condition in the prolapsed pelvic microenvironment, we tried to identify the overlaps of significantly differentially expressed genes enriched GO terms among these three prolapsed tissues. There were 67 up-regulated GO terms and 9 down-regulated GO terms overlapped in all tissues (Figure 3G, 3H). In particular, the top 3 significantly up-regulated GO terms are inflammatory response, positive regulation of cell proliferation and regulation of leukocyte activation. By comparison, the down-regulated GO terms involved in regulation of cell differentiation, regulation of development process and positive regulation of biological process. These results indicated a widespread inflammatory microenvironment and an abnormal cell biological process in the prolapsed pelvic floor.

### Loxl1 knockout mice mimic the phenotype and functional changes of clinical POP

**Figure 4:**
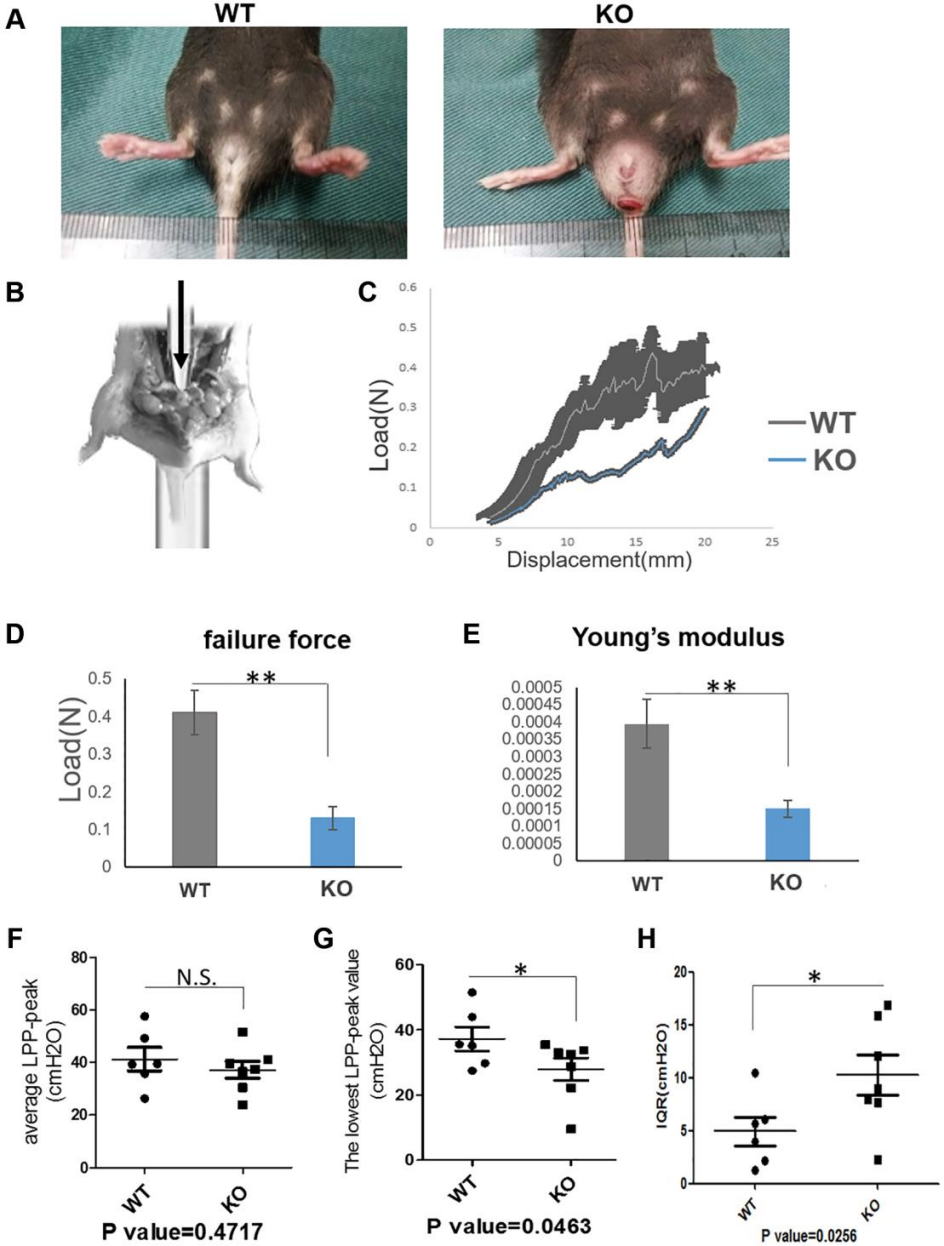
**Loxl1 knockout mice mimic the phenotype and functional changes of clinical POP.** (**A**) The gross pictures showed there was a pelvic organ prolapse phenotype in Loxl1 knockout mice. (**B**) The simulated image of pelvic floor support test. (**C**) The pelvic floor support test showed the different mechanical property of the pelvic floor from Loxl1 knockout mice and the wild type mice, horizontal axis represent the displacement (mm) and vertical axis represent the load (N), *n* = 4. (**D**, **E**) the failure force (**D**) and Young’s modulus (**E**) of pelvic floor showing that the values from the WT group were higher than the loxl1 knockout group, *n* = 4. (**F**–**H**) LPP was performed to test the urinary function, there were decreased average LPP-peak value (**F**) and decreased lowest LPP-peak value (**G**) while there was an increased Inter-Quartile Range (IQR) of the average LPP-peak value (**H**) in the Loxl1 knockout mice.

Loxl1 was directly involved in the synthesis and assembly of elastin [13]. It has been reported that over 50% of Loxl1 knockout mice can have a phenotype of vagina prolapse and rectum prolapse after pregnancy [11, 16]. 80% Loxl1 knockout mice can suffer from POP naturally in 17 months old even without production [18]. For this reason, we introduced the Loxl1 knockout mice and identified its phenotype and histological changes. From the gross view, the knockout mice had an obvious phenotype of POP indeed (Figure 4A).

On the respect of function evaluation of the knockout mice and the wild-type mice, we performed the pelvic floor support testing (Figure 4B) to evaluate the mechanical properties and performed LPP testing to evaluate the urinary function. Mechanical properties (failure force and Young’s modulus) of pelvic floor showed that the values from the WT group were significantly higher than the loxl1 knockout group (Figure 4C–4E). At the same time, average LPP-peak value and lowest LPP-peak value were both decreased. However, Inter-Quartile Range (IQR) of the average LPP-peak value in the Loxl1 knockout mice was increased (Figure 4F–4H). The average LPP-peak value and lowest LPP-peak value could represent the average pressure and lowest pressure threshold that the bladder can tolerate. The IQR of the average LPP-peak value could represent the functional stability of bladder under pressure. Taken together, these results demonstrated that loxl1 deficiency was associated with the reduction in mechanical property of pelvic floor and urinary function.

### Loxl1 knockout mice reproduce the micro-structural changes of clinical POP

**Figure 5:**
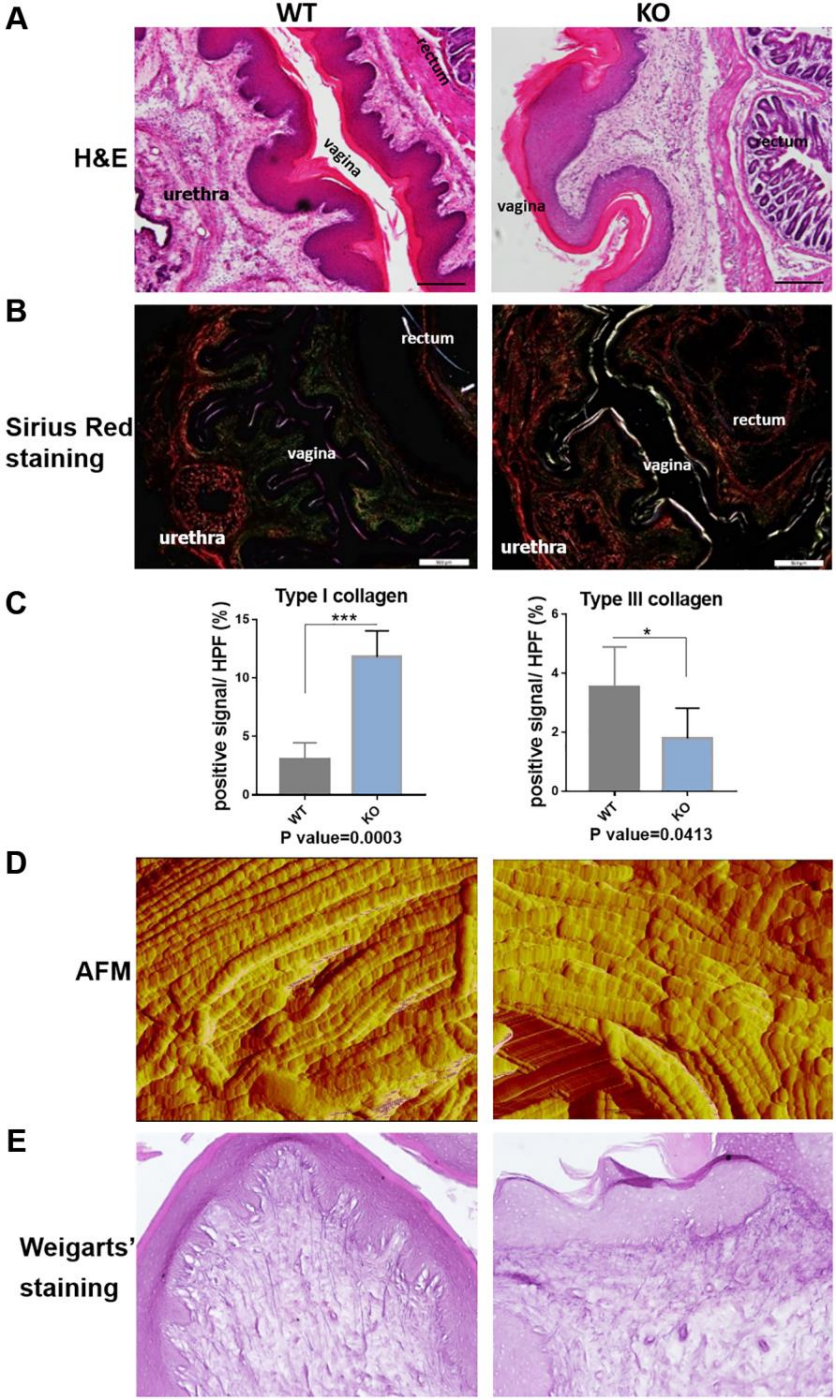
**Loxl1 knockout mice reproduce the micro-structural changes of clinical POP.** (**A**) HE staining showed obvious microstructure space and disappeared Vicious Angle structure in Loxl1 knockout mice, scale bar 100 um. (**B**) Sirius red staining demonstrated increased type I collagen and decreased type III collagen in Loxl1 knockout mice, scale bar 100 um. (**C**) Sirius red staining as in B was quantified, the proportion of positive signals area (red signals for type I collage and green signals for type III collagen) in each High Power Field of vision (HPF) were quantified, *n* = 4 in each group. (**D**) Atom force microscope (AFM) showed aligned collagen fibers in WT mice while disordered arranged collagen fibers in Loxl1 knockout mice. (**E**) Weigarts’ staining of the vagina tissue showed the elastic fibers in WT mice were linear and polarized, while in the knockout mice were fragmented and arranged disordered.

**Figure 6:**
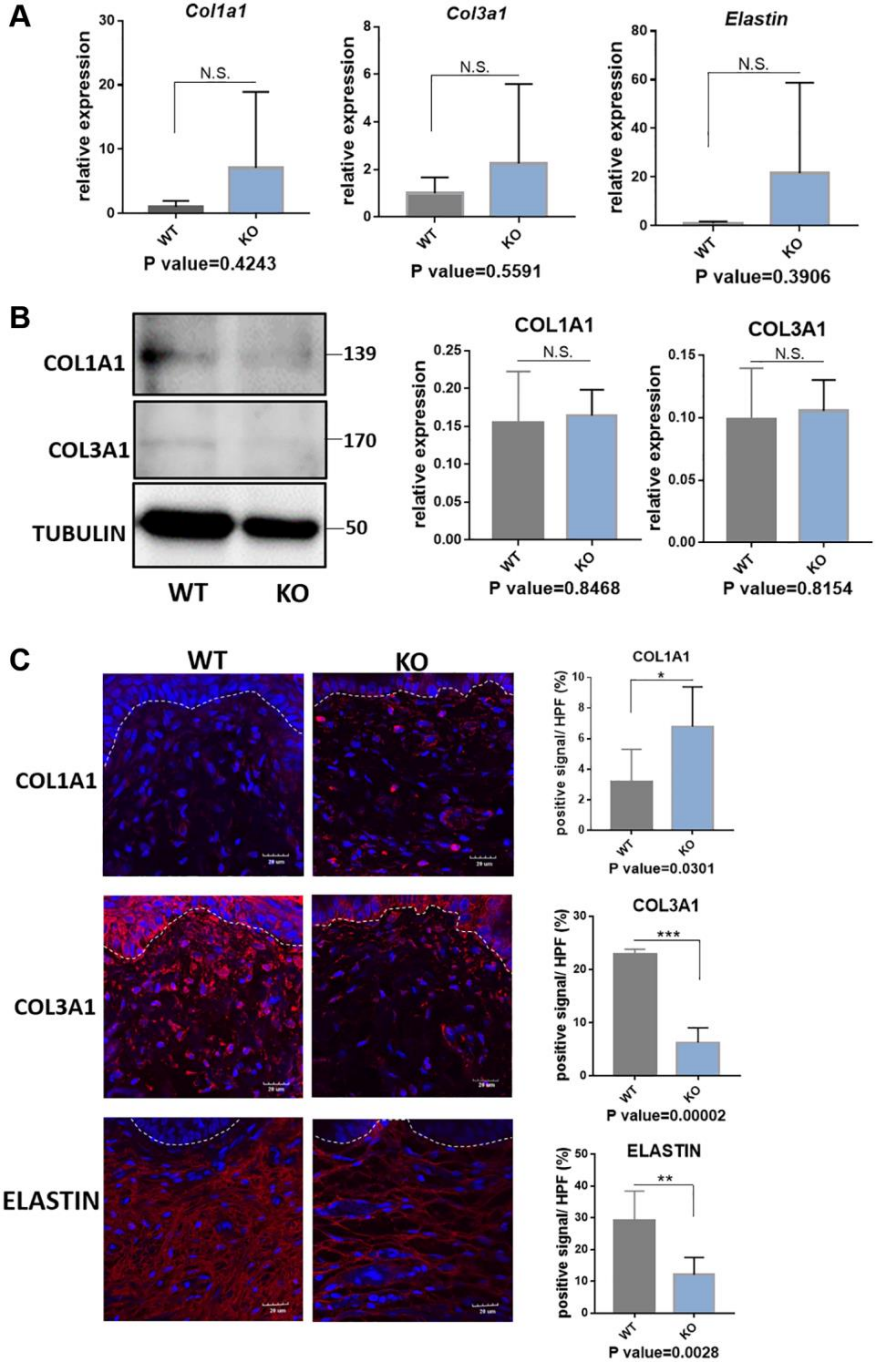
**Quantification analyses of type I collagen, type III collagen and elastin in vagina tissue of Loxl1 knockout mice.** (**A**) qRT-PCR analyses showed mRNA expression level of Col1a1, Col3a1 and Elastin in vagina tissue (WT: wild type mice, KO: Loxl1 knockout mice, *n* = 3 in each group). (**B**) Western blots analyses of COL1A1 and COL3A1 in vagina tissue. Relative content of proteins were quantified on the right, the levels of protein expressed relative to TUBULIN, (*n* = 3 in each group). (**C**) Immunofluorescence staining for visualization (red) of COL1A1, COL3A1 and ELASTIN in vagina tissue (White dotted line indicated the interface between mucus layer and submucosa layer). The proportion of positive signal area (red) in each High Power Field of vision (HPF) of each protein were quantified on the right, (*n* = 6 in each group).

In the following, we performed the histological staining to confirm the structural changes in mice model. H&E staining images showed that the vaginal circular muscle layer became obvious thinner (Figure 5A). Sirius red staining further revealed type III collagen fibers in the submucosal layer of vagina were reduced in the knockout mice. On the contrary, the content of type I collagen fibers in red color was increased (Figure 5B). Quantification result of Sirius staining showed significantly in increased type I collagen and decreased type III collagen in KO mice (Figure 5C). The changes of collagen could be further proved through western blot analyses and immunofluorescence staining (Figure 6B and 6C), although the qRT-PCR analyses showed relatively increased mRNA expression both of Col1a1 and Col3a1 in KO group of animal samples (Figure 6A). To observe the arrangement of collagen bundles, we performed the atomic force microscope (AFM) of the vagina tissue. Collagen fibers in wild-type mice were mostly arranged regularly and orderly compared with Loxl1 knockout mice (Figure 5D). The Weigarts’ staining also confirmed that the elastic fibers in wild-type mice were linear and polarized, while it is fragmented and disordered in knockout mice (Figure 5E). Immunofluorescence staining for elastin also showed diffuse and weak in knockout mice, suggestive of reduced elastin polymer deposition (Figure 6C). Importantly, these changes were consistent with the histological changes of human samples, indicating the possibility of simulating POP in human.

### Loxl1 knockout mice recapitulate the molecular changes in vagina tissue of clinical POP

**Figure 7:**
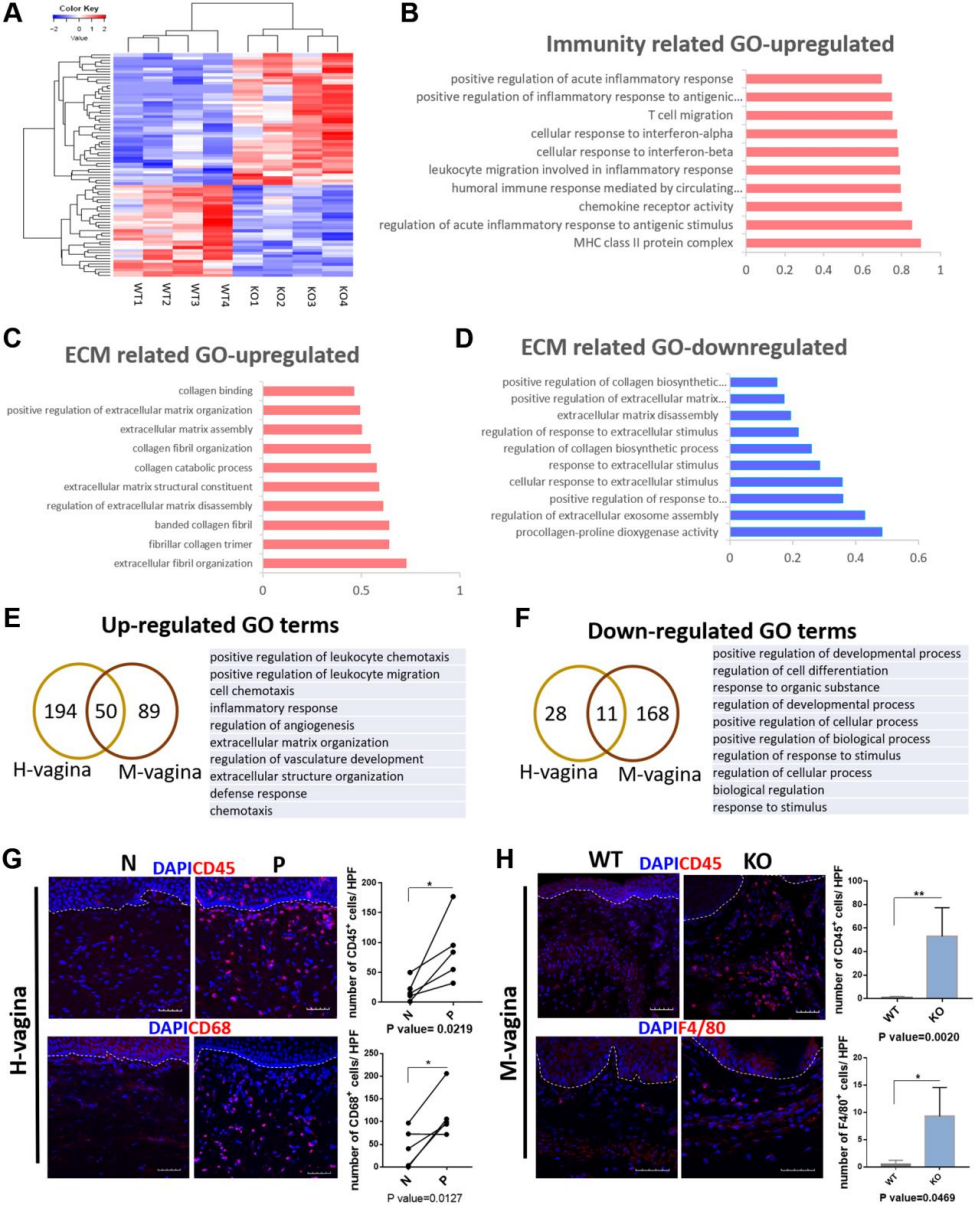
**Loxl1 knockout mice recapitulate the molecular changes in vagina tissue of clinical POP.** (**A**) Heat map across all the samples using the top 500 most differently expressed genes in vagina tissue between the Loxl1 knockout mice and WT mice. (**B**–**D**) gene ontology categories of differently expressed genes, horizontal axis represent the fold enrichment, *p* value < 0.05. (**E**, **F**) Venn diagram (left) and the form (right) showed the overlaps of up-regulated (**E**) and down-regulated (**F**) differentially expressed genes enriched GO terms between human vagina (H-vagina) and mice vagina (M-vagina). (**G**, **H**) Immunofluorescence staining for visualization (red) of immune cell infiltration in human vagina (**G**) and mice vagina (**H**). The number of positive cells (red) in each High Power Field of vision (HPF) were quantified respectively. (CD45: leukocyte, CD68: macrophage in human, F4/80: macrophage in mice; White dotted line indicated the interface between mucus layer and submucosa layer, *n* = 5 in each group).

To further explore whether the molecular changes in Loxl1 knockout mice have the similar consistence with clinical POP patients. We performed mRNA-sequencing analysis by comparing the vagina tissue of Loxl1 knockout mice with wild-type mice. The heat map of differently expressed genes show that the samples from Loxl1 knockout mice and wild-type mice could be clearly separated and clustered into two groups, indicating little variation in each group (Figure 7A). Similar to the results of human samples, there was also a group of up-regulated immune related GO terms (Figure 7B), including positive regulation of acute inflammatory response, T cell migration, leukocyte migration, cellular response to interferon-alpha and interferon-beta. The increased ECM related GO terms mainly focused on ECM organization and catabolism process, including ECM and collagen assembly and collagen catabolism process (Figure 7C). While the down-regulated ECM related GO terms focused on the synthetic process, such as regulation of collagen biosynthetic process, ECM disassembly and positive regulation of ECM organization (Figure 7D). Furthermore, by depicting the overlapped differentially expressed genes enriched GO terms between human vagina and mice vagina tissues, they were found to share some common terms (Figure 7E, 7F). The commonly up-regulated GO terms mainly focused on immune response and ECM organization, including positive regulation of leukocyte chemotaxis, positive regulation of leukocyte migration, inflammation response and extracellular matrix organization, extracellular structure organization. Immunofluorescence staining of leukocyte marker (CD45) and macrophage maker (CD68 in human sample and F4/80 in mice sample) showed significantly increased number of CD45 positive cells, CD68 positive cells, and F4/80 positive cells cell in prolapsed tissue of human samples and in KO group of animal samples, indicating the local cell infiltration of leukocyte and macrophage (Figure 7G, 7H). The verified results coincided well with transcriptomic changes with up-regulated GO terms related to positive regulation of leukocyte migration and inflammation response in both prolapsed tissue of human samples and KO group of animal samples. The commonly down-regulated GO terms mainly involved in cellular activities, like positive regulation of developmental process, regulation of cell differentiation and biological process. These results confirmed the same pathological changes in transcriptomic and molecular level between human with POP and Loxl1 knockout mice.

## DISCUSSION

The onset and progression of POP is a multifactorial and complex process, which is related to the destruction of the integrity of pelvic support tissue in structure. The elastic fiber is one of the major components of pelvic support tissue and enable the pelvic support tissues with scalability and reversible deformation capacity. Drewes suggested the metabolism imbalance of elastic fiber was considered as the fundamental pathogenesis of POP [10]. Lately, Goepel et al. confirmed that the elastin maintained integrity in the sacral ligaments of healthy postmenopausal women, while the elastin was damaged or even missing in the patients with POP [26]. In addition to the elastin, collagen is also the main components in connective tissues and attaches scalability to these tissues. Type I collagen has no elastic properties, but gives the tissue strong tensile properties, while type III collagen has elastic properties and is abundant in soft tissue [27]. And the previous study stated that a change in the type I to type III collagen ratio are typical of older tissue [28]. Such remodeling and structural changes of the main components of ECM may contribute to the decreased elasticity and mechanical property of the connective tissue. Consistent with these outcomes, our results indicated loss of elastic fibers, decreased expression of elastin content as well as an increased ratio of collagen I/collagen III, may play a crucial role in the development of POP.

AFM is able to observe the diameter of a single collagen fibril and the structure of each D-period [29]. The ultrastructure determines the toughness and tensile resistance of collagen fibers [30]. The results of AFM observing the vagina tissue of Loxl1 knockout mice found disordered bundles of collagen fiber to further confirm the abnormal ECM in the vaginal tissue of Loxl1 knockout mice. Besides the structural changes of pelvic support tissue in POP patients, there are severely functional changes of pelvic organs. 40%~50% of women with POP suffered from stress urinary incontinence [31, 32], and the clinical retrospective study suggested the relationship between POP and decreased LPP [33]. Hence, we assessed the urinary function by measuring the LPP. The measurement of LPP truly showed a decreased urinary function in Loxl1 knockout mice. The results were consistent with the published studies [11, 19]. We also performed the pelvic support testing to directly reflect the mechanical property of pelvic support tissue. It is interesting that Loxl1 knockout mice had a decreased mechanism property of the pelvic floor. All results indicated either Loxl1 knockout mice model or clinical patients gave the similar histo-pathological and functional changes which involved in the pathogenesis of POP.

Loxl1 belongs to the Lysyl oxidase family. Like the other member of this family, the molecular structure of Loxl1 has a highly conserved C-terminal domain that includes a copper-binding motif, a lysyl-tyrosine-quinone cofactor and cytokine receptor-like domain [34]. The lysyl-tyrosine-quinone cofactor is required for lysyl oxidase oxidation of its primary amine substrates: the epsilon amino group of lysine or hydroxylysine residues in collagens and lysine residues in elastin [35]. Among the Lox family members, only Loxl1 levels is coincident with the expression of crosslinked or insoluble elastin [36]. In the elastin crosslinking process, the oxidative deamination of lysine residues in tropoelastin is catalyzed by loxl1 to α-aminoadipic acid δ-semialdehyde, which can spontaneously condense with neighboring amino groups or other peptidyl aldehydes to form covalent cross-links [37]. The function of elastin is conferring elasticity and resilience to connective tissues, such as blood vessel walls, lung, skin, trabecular meshwork and female reproductive tissues [38, 39]. Due to the biological function of Loxl1 on elastin, deficiency of Loxl1 underlie a number of pathological processes related to an imbalance in ECM metabolism. In our previous study, Loxl1 deficiency can effectively result in a different body-wide transcriptomic map of 17 organs from Loxl1 knockout mice and wild type mice [40]. In vagina, it has also been proved that Loxl1 deficiency can influence the mRNA expression levels of key matrix metalloproteinases (Mmp2, Mmp9, Mmp12), tissue inhibitors of metalloproteinases (Timp1, Timp2, Timp3, Timp4), and ECM components including collagen type 1 (Col1a1), collagen type 3 (Col3a1), fibulin 5 (Fbln5), alpha smooth muscle actin (Acta2) [41]. Importantly, the transcriptomic changes of POP patients and mice model further confirmed the abnormal ECM homeostasis in the molecular level. As a key factor of POP pathogenesis, GO analysis showed an abnormal synthesis and metabolism of ECM in clinical patients compared to the control group. The up-regulated GO (related to ECM catabolism) and down-regulated GO (related to ECM synthesis) were existed in the vagina tissue of Loxl1 knockout mice, showing great similarity with the results from clinical patients. Notably, an abnormal ECM homeostasis in the pelvic floor was also characterized in Loxl1 knockout mice, meaning the mice model could well simulate the ECM molecular pathological microenvironment in human POP.

On the other hand, there were a variety of immune related GO terms up-regulated in multiple pelvic floor tissues of POP patients and mice model, indicating a general inflammatory environment in the pelvic floor. Potentially more relevant to our results is the finding by BING ZHAO et al. showing a high mRNA expression of IFNγ, IFNGR and IFNGR2 in the uterine tissues of POP patients, and the mRNA expression of genes involved in signaling pathway JAK-STAT was up-regulated which participate in the activation of IFNγ [42]. In addition, Yeo Jung Moon and Sang Wook Bai et al. found a high expression of IL5 in the sacral ligament in POP patient [43]. Though there were no existing evidence proved that Loxl1 can direct regulate the inflammatory cytokines secretion, according to the association analysis, Loxl1 was significantly associated with intratumoral inflammation and interleukin-4 expression level in frozen tumor tissues [44]. In Pseudoexfoliation Glaucoma, the deficiency of Loxl1 is associated with decreased elastin incorporation into elastic lamina of blood vessels, resulting in released soluble elastin and leakage of serum proteins, inflammatory cytokines into aqueous humor [39]. The inflammatory cytokines leakage process happened in Pseudoexfoliation Glaucoma may fit to the up-regulated inflammatory response in pelvic tissues. Based on the pathological changes in POP patients, it can be concluded that the vagina tissue from POP patients had a disrupted microstructure of ECM fibers. And multiple pelvic tissues (including vagina, round ligament and Uterosacral ligament) had a high level of inflammatory response. These results may put forward a molecular mechanism of POP. According to the previous studies, immune and inflammatory cells can engage in complex interplay with resident non-immune cells and the ECM of tissue during tissue injury [45, 46]. The ECM and the immune system are intertwined, which may promote ECM to exacerbate these pathological conditions through increased and sustained inflammation. This may eventually lead to the development of new angles or approaches that can reverse the pelvic inflammatory condition for clinical POP treatment.

To the best of our knowledge, this study comprehensively investigated the role of Loxl1 in POP pathology. The novel finding on the correlation between human and animal models has provided the similar pathological profiles in either POP patients or Loxl1 knockout mice, including prolapsed phenotypes, histological changes, functional changes and genomic changes. The strengths of this study include the analysis of POP molecular pathology by combining multiple tissues in pelvic floor, giving the common pathological changes in pelvic floor tissues. This study compared the pathological changes happened in tissues from POP patients and Loxl1 knockout mice from histological, functional to molecular level, and verified the similarity between POP patients and Loxl1 knockout mice.

## CONCLUSIONS

This study provided evidences for deeply understanding the pathological of POP via Lox1 knockout model, confirming that the onset of POP was not only associated with the abnormal metabolism of ECM, but also related to widespread changes of inflammatory environment in the pelvic floor. On the other hand, this study systematically explored the similarity of Loxl1 knockout mice and POP patients, providing a promising animal model for exploring the treatment and pathogenesis of POP.

## References

[1] Smith P, Willemsen D, Popkes M, Metge F, Gandiwa E, Reichard M, Valenzano DR. Regulation of life span by the gut microbiota in the short-lived African turquoise killifish. Elife. 2017; 6:e27014. 10.7554/eLife.27014 28826469 PMC5566455

[2] Abdel-Fattah M, Familusi A, Fielding S, Ford J, Bhattacharya S. Primary and repeat surgical treatment for female pelvic organ prolapse and incontinence in parous women in the UK: a register linkage study. BMJ Open. 2011; 1:e000206. 10.1136/bmjopen-2011-000206 22102637 PMC3221293

[3] Deprest J, Feola A. The need for preclinical research on pelvic floor reconstruction. BJOG. 2013; 120:141–3. 10.1111/1471-0528.12088 23240796

[4] Couri BM, Lenis AT, Borazjani A, Paraiso MF, Damaser MS. Animal models of female pelvic organ prolapse: lessons learned. Expert Rev Obstet Gynecol. 2012; 7:249–60. 10.1586/eog.12.24 22707980 PMC3374602

[5] Abramowitch SD, Feola A, Jallah Z, Moalli PA. Tissue mechanics, animal models, and pelvic organ prolapse: a review. Eur J Obstet Gynecol Reprod Biol. 2009 (Suppl 1); 144:S146–58. 10.1016/j.ejogrb.2009.02.022 19285776

[6] Otto LN, Slayden OD, Clark AL, Brenner RM. The rhesus macaque as an animal model for pelvic organ prolapse. Am J Obstet Gynecol. 2002; 186:416–21. 10.1067/mob.2002.121723 11904600

[7] Schimpf M, Tulikangas P. Evolution of the female pelvis and relationships to pelvic organ prolapse. Int Urogynecol J Pelvic Floor Dysfunct. 2005; 16:315–20. 10.1007/s00192-004-1258-1 15654501

[8] Ulrich D, Edwards SL, Su K, White JF, Ramshaw JA, Jenkin G, Deprest J, Rosamilia A, Werkmeister JA, Gargett CE. Influence of reproductive status on tissue composition and biomechanical properties of ovine vagina. PLoS One. 2014; 9:e93172. 10.1371/journal.pone.0093172 24709913 PMC3977844

[9] Connell KA, Guess MK, Chen H, Andikyan V, Bercik R, Taylor HS. HOXA11 is critical for development and maintenance of uterosacral ligaments and deficient in pelvic prolapse. J Clin Invest. 2008; 118:1050–5. 10.1172/JCI34193 18274672 PMC2242622

[10] Drewes PG, Yanagisawa H, Starcher B, Hornstra I, Csiszar K, Marinis SI, Keller P, Word RA. Pelvic organ prolapse in fibulin-5 knockout mice: pregnancy-induced changes in elastic fiber homeostasis in mouse vagina. Am J Pathol. 2007; 170:578–89. 10.2353/ajpath.2007.060662 17255326 PMC1851882

[11] Lee UJ, Gustilo-Ashby AM, Daneshgari F, Kuang M, Vurbic D, Lin DL, Flask CA, Li T, Damaser MS. Lower urogenital tract anatomical and functional phenotype in lysyl oxidase like-1 knockout mice resembles female pelvic floor dysfunction in humans. Am J Physiol Renal Physiol. 2008; 295:F545–55. 10.1152/ajprenal.00063.2008 18495804

[12] McLaughlin PJ, Bakall B, Choi J, Liu Z, Sasaki T, Davis EC, Marmorstein AD, Marmorstein LY. Lack of fibulin-3 causes early aging and herniation, but not macular degeneration in mice. Hum Mol Genet. 2007; 16:3059–70. 10.1093/hmg/ddm264 17872905

[13] Liu X, Zhao Y, Gao J, Pawlyk B, Starcher B, Spencer JA, Yanagisawa H, Zuo J, Li T. Elastic fiber homeostasis requires lysyl oxidase-like 1 protein. Nat Genet. 2004; 36:178–82. 10.1038/ng1297 14745449

[14] Klutke J, Ji Q, Campeau J, Starcher B, Felix JC, Stanczyk FZ, Klutke C. Decreased endopelvic fascia elastin content in uterine prolapse. Acta Obstet Gynecol Scand. 2008; 87:111–5. 10.1080/00016340701819247 18158636

[15] Zhao BH, Zhou JH. Decreased expression of elastin, fibulin-5 and lysyl oxidase-like 1 in the uterosacral ligaments of postmenopausal women with pelvic organ prolapse. J Obstet Gynaecol Res. 2012; 38:925–31. 10.1111/j.1447-0756.2011.01814.x 22487196

[16] Couri BM, Borazjani A, Lenis AT, Balog B, Kuang M, Lin DL, Damaser MS. Validation of genetically matched wild-type strain and lysyl oxidase-like 1 knockout mouse model of pelvic organ prolapse. Female Pelvic Med Reconstr Surg. 2014; 20:287–92. 10.1097/SPV.0000000000000104 25181380 PMC4155759

[17] Gustilo-Ashby AM, Lee U, Vurbic D, Sypert D, Kuang M, Daneshgari F, Barber MD, Damaser MS. The impact of cesarean delivery on pelvic floor dysfunction in lysyl oxidase like-1 knockout mice. Female Pelvic Med Reconstr Surg. 2010; 16:21–30. 10.1097/SPV.0b013e3181d00035 22453086

[18] Alperin M, Debes K, Abramowitch S, Meyn L, Moalli PA. LOXL1 deficiency negatively impacts the biomechanical properties of the mouse vagina and supportive tissues. Int Urogynecol J Pelvic Floor Dysfunct. 2008; 19:977–86. 10.1007/s00192-008-0561-7 18265927 PMC3037182

[19] Liu G, Daneshgari F, Li M, Lin D, Lee U, Li T, Damaser MS. Bladder and urethral function in pelvic organ prolapsed lysyl oxidase like-1 knockout mice. BJU Int. 2007; 100:414–8. 10.1111/j.1464-410X.2007.06929.x 17555473

[20] Liu X, Zhao Y, Pawlyk B, Damaser M, Li T. Failure of elastic fiber homeostasis leads to pelvic floor disorders. Am J Pathol. 2006; 168:519–28. 10.2353/ajpath.2006.050399 16436666 PMC1606509

[21] Shi LB, Cai HX, Chen LK, Wu Y, Zhu SA, Gong XN, Xia YX, Ouyang HW, Zou XH. Tissue engineered bulking agent with adipose-derived stem cells and silk fibroin microspheres for the treatment of intrinsic urethral sphincter deficiency. Biomaterials. 2014; 35:1519–30. 10.1016/j.biomaterials.2013.11.025 24275524

[22] Zhang S, Jiang YZ, Zhang W, Chen L, Tong T, Liu W, Mu Q, Liu H, Ji J, Ouyang HW, Zou X. Neonatal desensitization supports long-term survival and functional integration of human embryonic stem cell-derived mesenchymal stem cells in rat joint cartilage without immunosuppression. Stem Cells Dev. 2013; 22:90–101. 10.1089/scd.2012.0116 22788986 PMC3528094

[23] Argyropoulos AJ, Robichaud P, Balimunkwe RM, Fisher GJ, Hammerberg C, Yan Y, Quan T. Alterations of Dermal Connective Tissue Collagen in Diabetes: Molecular Basis of Aged-Appearing Skin. PLoS One. 2016; 11:e0153806. 10.1371/journal.pone.0153806 27104752 PMC4841569

[24] Liu Z, Li X, Zhang JT, Cai YJ, Cheng TL, Cheng C, Wang Y, Zhang CC, Nie YH, Chen ZF, Bian WJ, Zhang L, Xiao J, et al. Autism-like behaviours and germline transmission in transgenic monkeys overexpressing MeCP2. Nature. 2016; 530:98–102. 10.1038/nature16533 26808898

[25] Brizzolara SS, Killeen J, Urschitz J. Gene expression profile in pelvic organ prolapse. Mol Hum Reprod. 2009; 15:59–67. 10.1093/molehr/gan074 19056808 PMC2639232

[26] Goepel C. Differential elastin and tenascin immunolabeling in the uterosacral ligaments in postmenopausal women with and without pelvic organ prolapse. Acta Histochem. 2008; 110:204–9. 10.1016/j.acthis.2007.10.014 18155129

[27] Ramshaw JA, Peng YY, Glattauer V, Werkmeister JA. Collagens as biomaterials. J Mater Sci Mater Med. 2009 (Suppl 1); 20:S3–8. 10.1007/s10856-008-3415-4 18379858

[28] Jackson SR, Avery NC, Tarlton JF, Eckford SD, Abrams P, Bailey AJ. Changes in metabolism of collagen in genitourinary prolapse. Lancet. 1996; 347:1658–61. 10.1016/s0140-6736(96)91489-0 8642960

[29] Rigozzi S, Stemmer A, Müller R, Snedeker JG. Mechanical response of individual collagen fibrils in loaded tendon as measured by atomic force microscopy. J Struct Biol. 2011; 176:9–15. 10.1016/j.jsb.2011.07.002 21771659

[30] Rigozzi S, Müller R, Stemmer A, Snedeker JG. Tendon glycosaminoglycan proteoglycan sidechains promote collagen fibril sliding-AFM observations at the nanoscale. J Biomech. 2013; 46:813–8. 10.1016/j.jbiomech.2012.11.017 23219277

[31] Chen B, Wen Y, Polan ML. Elastolytic activity in women with stress urinary incontinence and pelvic organ prolapse. Neurourol Urodyn. 2004; 23:119–26. 10.1002/nau.20012 14983422

[32] Dietz HP, Hansell NK, Grace ME, Eldridge AM, Clarke B, Martin NG. Bladder neck mobility is a heritable trait. BJOG. 2005; 112:334–9. 10.1111/j.1471-0528.2004.00428.x 15713150

[33] Whiteside JL, Viazmenski A, Strohbehn K, Hanissian PD. Does pelvic organ prolapse reduction affect abdominal leak point pressures? J Reprod Med. 2008; 53:294–8. 18472654

[34] Greene AG, Eivers SB, Dervan EWJ, O'Brien CJ, Wallace DM. Lysyl Oxidase Like 1: Biological roles and regulation. Exp Eye Res. 2020; 193:107975. 10.1016/j.exer.2020.107975 32070696

[35] Trackman PC. Lysyl Oxidase Isoforms and Potential Therapeutic Opportunities for Fibrosis and Cancer. Expert Opin Ther Targets. 2016; 20:935–45. 10.1517/14728222.2016.1151003 26848785 PMC4988797

[36] Zhao W, Yang A, Chen W, Wang P, Liu T, Cong M, Xu A, Yan X, Jia J, You H. Inhibition of lysyl oxidase-like 1 (LOXL1) expression arrests liver fibrosis progression in cirrhosis by reducing elastin crosslinking. Biochim Biophys Acta Mol Basis Dis. 2018; 1864:1129–37. 10.1016/j.bbadis.2018.01.019 29366776

[37] Sato F, Seino-Sudo R, Okada M, Sakai H, Yumoto T, Wachi H. Lysyl Oxidase Enhances the Deposition of Tropoelastin through the Catalysis of Tropoelastin Molecules on the Cell Surface. Biol Pharm Bull. 2017; 40:1646–53. 10.1248/bpb.b17-00027 28966236

[38] Schlötzer-Schrehardt U, Zenkel M. The role of lysyl oxidase-like 1 (LOXL1) in exfoliation syndrome and glaucoma. Exp Eye Res. 2019; 189:107818. 10.1016/j.exer.2019.107818 31563608

[39] Vazquez LE, Lee RK. Genomic and proteomic pathophysiology of pseudoexfoliation glaucoma. Int Ophthalmol Clin. 2014; 54:1–13. 10.1097/IIO.0000000000000047 25171640 PMC4182319

[40] Li Y, Wu B, An C, Jiang D, Gong L, Liu Y, Liu Y, Li J, Ouyang H, Zou X. Mass cytometry and transcriptomic profiling reveal body-wide pathology induced by Loxl1 deficiency. Cell Prolif. 2021; 54:e13077. 10.1111/cpr.13077 34105806 PMC8249785

[41] Borazjani A, Couri BM, Kuang M, Balog BM, Damaser MS. Role of lysyl oxidase like 1 in regulation of postpartum connective tissue metabolism in the mouse vagina†. Biol Reprod. 2019; 101:916–27. 10.1093/biolre/ioz148 31403161 PMC7245155

[42] Zhao B, Yan J, Wu H, Zhou Y, Xu D, Hu M, Cui S. Interferon-γ and its pathway-associated gene expression in the vaginal tissue of premenopausal females with pelvic organ prolapse. Exp Ther Med. 2014; 8:1145–9. 10.3892/etm.2014.1868 25187813 PMC4151656

[43] Moon YJ, Bai SW, Jung CY, Kim CH. Estrogen-related genome-based expression profiling study of uterosacral ligaments in women with pelvic organ prolapse. Int Urogynecol J. 2013; 24:1961–7. 10.1007/s00192-013-2124-9 23700042

[44] Jeong YJ, Park SH, Mun SH, Kwak SG, Lee SJ, Oh HK. Association between lysyl oxidase and fibrotic focus in relation with inflammation in breast cancer. Oncol Lett. 2018; 15:2431–40. 10.3892/ol.2017.7617 29434955 PMC5777281

[45] Tomlin H, Piccinini AM. A complex interplay between the extracellular matrix and the innate immune response to microbial pathogens. Immunology. 2018; 155:186–201. 10.1111/imm.12972 29908065 PMC6142291

[46] Hallmann R, Zhang X, Di Russo J, Li L, Song J, Hannocks MJ, Sorokin L. The regulation of immune cell trafficking by the extracellular matrix. Curr Opin Cell Biol. 2015; 36:54–61. 10.1016/j.ceb.2015.06.006 26189064

